# Advancements in robotic surgery for vitreoretinal diseases: current trends and the future

**DOI:** 10.1007/s10384-025-01231-1

**Published:** 2025-08-01

**Authors:** Shintaro Nakao, Kotaro Tadano, Koh-Hei Sonoda

**Affiliations:** 1 Department of Ophthalmology, Juntendo University Graduate School of Medicine, Tokyo, Japan; 2 Institute of Innovation Research, Institute of Science Tokyo, Yokohama, Japan; 3 Department of Ophthalmology, Graduate School of Medical Science, Kyushu University, Kyushu University, 3-1-1 Maidashi, Higashi-Ku, Fukuoka, 812-8582 Japan

**Keywords:** Robotics, Vitreoretinal surgery, Operation assistance robot, Observation robot

## Abstract

Medical robotics, such as the da Vinci Surgical System, Hinotori, and Saroa, has been rapidly expanding in the field of general surgery in recent years. Because of the need for high precision in vitreoretinal surgery, general surgery robots are not applicable; therefore, a variety of robots specifically designed for vitreoretinal surgery have been developed around the world. These robotic systems can be broadly categorized into operation systems, operation assistance systems, and observation systems. The purpose of the operation robots is mainly internal limiting membrane peeling and retinal vascular cannulation. The PRECEYES Surgical System was already approved in the European Union in 2019 on the basis of positive clinical results. The aim of operation assistance robots, such as iARMs, is to suppress tremors. Meanwhile, we have developed an observation robot, an intraocular endoscope-holding robot (OQrimo), which was approved as a medical device in Japan in 2023. This robot allows surgeons to perform intraocular manipulations using both hands during vitreoretinal surgery for proliferative diabetic retinopathy and other intractable retinal diseases and may also make it easier to manipulate the anterior tissues around the ciliary body. In the future, by being combined with artificial intelligence in vitreoretinal clinics, robotic surgery will be applied to preoperative, intraoperative, and postoperative surgical procedure decisions, programs, and education.

## Introduction

### Current status of vitreoretinal surgery

**Fig. 1:**
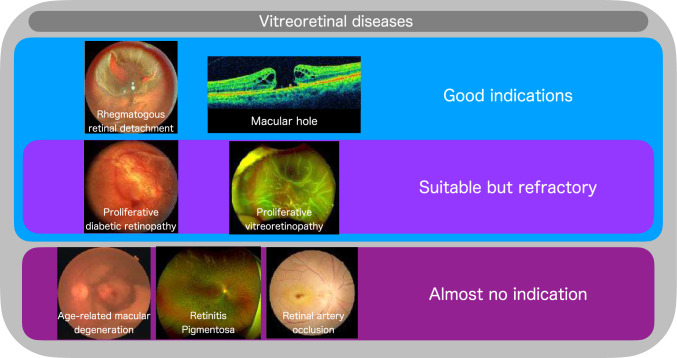
Various vitreoretinal diseases and indications for vitrectomy. Currently, rhegmatogenous retinal detachment and macular hole are considered good indications for vitrectomy, and the treatment outcomes are generally good. However, some diseases, such as proliferative diabetic retinopathy and proliferative vitreous retinopathy, are suitable for vitrectomy but difficult to treat. Additionally, age-related macular degeneration, retinitis pigmentosa, and retinal artery occlusion are not suitable for vitrectomy. However, gene therapy, cell therapy, and intravascular therapy may be possible for these disorders in the future, and precise vitrectomy will be essential

The treatment of vitreoretinal diseases has been undergoing major changes in the last 20 years. In Japan, diabetic retinopathy and cataracts were the leading causes of blindness in 1991, but the number of cases with blindness has decreased dramatically [1]. One of the positive factors behind this is the evolution of ophthalmic surgeries. In particular, the progress in vitreoretinal surgery has been remarkable. With wide-viewing systems and closure valve trocar systems, minimally invasive vitrectomy surgery (MIVS) has become widely used in the ophthalmology field [2]. As a result, the success rates of vitrectomy for macular epiretinal membrane (ERM), macular hole, and rhegmatogenous retinal detachment have improved remarkably [3–5] (Fig. 1). Although the current types of vitreoretinal surgery are well defined, certain severe conditions such as proliferative vitreoretinopathy (PVR) and proliferative diabetic retinopathy (PDR) can pose a significant challenge even for experienced surgeons [6]. Vitreoretinal surgery demands higher precision while dealing with the fragile neural tissue of the retina.

On the other hand, in Japan, the data on blindness in 2017 showed an increase in retinal degenerative diseases such as glaucoma, retinitis pigmentosa, age-related macular degeneration, and retinal and choroidal atrophy including myopia [1]. These diseases and disorders are primarily treatable by drugs or have no cure, and the indications for vitreoretinal surgery are currently limited (Fig. 1). In recent years, however, gene therapy, cell therapy, and regenerative medicine have been expected to be effective against these conditions [7–9]. Successful vitreoretinal surgery is essential for these promising new treatments. Furthermore, although no definitive treatment exists for rare diseases such as retinal artery occlusion, the effectiveness of endovascular treatment via vitrectomy has been reported [10]. These procedures require extremely precise techniques but carry limitations in terms of human hands’ manipulation, such as tremors and accuracy.

### Robotics in our lives

The concept of robots to create artificial humans has existed for a long time [11]. However, practical robots only appeared after the 1940s. Charlie Chaplin, in his film *Modern Times*, focused on mechanized civilization and predicted the coming of an era in which we coexist with robots [12]. Since then, remarkable development has been seen in fields such as space development and deep-sea exploration. At the same time, industrial robots have become indispensable to the economic development of recent years. These have made a great contribution to improvement of quality and human safety. And with the advent of an aging society facing developed countries, robotics are expected to further contribute to alleviation of the decline in the working population [12].

Robotics began to be used in the medical field in the 1980s [13], and the first telesurgical laparoscopic cholecystectomy was performed in 1997 [14]. Recently, robotic support in surgery has been significantly advanced, particularly in urological, gastrointestinal, and cardiovascular procedures [15]. The da Vinci Surgical System (Intuitive Surgical), released over 20 years ago, paved the way for innovations in this field. More recently, systems like the Hinotori (Medicaroid) and the Saroa (Riverfield), which incorporate physical feedback in forceps, have emerged in Japan [16]. These surgical support robots have high-performance cameras, providing surgeons with 3-dimensional vivid images during operations. Moreover, their multijoint functionality enables smooth access to traditionally challenging areas for laparoscopic techniques. Additionally, with stabilization technology, these robots facilitate precise surgical maneuvers. Given these advancements, the use of surgical support robots in medical treatment is expected to rise steadily.

For microsurgery, such as ophthalmic surgery, surgical robots for general surgery cannot be applied owing to differences in forceps size and required range of motion, although some reports of its application in external ophthalmic surgery have been published [17]. In addition, the accuracy of the da Vinci Surgical System is reported to be approximately 1 mm, making its application to microsurgery difficult [18]. Furthermore, responding to sudden patient movements is crucial because ophthalmic surgery is predominantly performed under local anesthesia. Addressing these problems requires the development of “ophthalmic surgery-specific robots,” and indeed, various ophthalmic surgery robots are being developed to confront these issues [19]. Robot-assisted surgery can potentially assist many vitreoretinal surgeons in treating the above-mentioned severe conditions more effectively and may also disclose new possibilities for vitreoretinal surgeries. The advantages of robot-assisted surgery lie in its tremor-free, high-precision movements, which result in low invasiveness and safety and potentially improve postoperative prognosis. Development research for robotic vitreoretinal surgery is being conducted worldwide, with cutting-edge systems already in use with actual patients. This paper summarizes the historical and current progress in robotic vitreoretinal surgery and outlines its prospects.

## History of vitreoretinal surgery robot development

The first attempts to apply micromanipulators to ophthalmic surgery were made in the 1950s [20]. Later, attempts to apply them to vitrectomy were made in the 1970s, but these devices were simple and limited to movement in 1 axis [21, 22]. Full-scale robotic vitrectomy surgery devices were reported in the 1980s. Spitznas reported an attempt at vitrectomy microsurgery with a motorized device [23]. This device had 1 arm that could hold various instruments, and the surgeon operated it with a joystick. Intuitive Surgical, which developed the da Vinci Surgical System, was founded in 1995, but attempts at using robots for intraocular surgery had already been proposed in 1997 [24]. On the basis of a concept similar to that of the da Vinci Surgical System, a joint team of the California Institute of Technology and Dr Steve Charles has developed a robotic system called Robot Assisted MicroSurgery (RAMS), which consists of 4 subsystems, including a slave robot and a master robot [24]. This system, called a telerobotic system, is controlled by the surgeon’s hands and foot switches, and has successfully grasped particles with a diameter of 0.015 inch (≈ 380 µm) inside a stimulated eye [24]. In Japan, Ueta and colleagues developed a prototype of a surgical robot to assist in vitrectomy surgery and conducted trials in 2009 [25]. They reported that the positioning accuracy (33.5 µm) was superior to manual surgery in porcine eyes. They also demonstrated that the robot was useful for creating posterior vitreous detachment and injecting drugs into retinal blood vessels with a diameter of approximately 80 µm. Sakai and colleagues developed a master-slave miniature robot for eye surgery [26]. However, these robots for vitreoretinal surgeries have not yet reached the stage of application in humans. In 2018, Edwards and colleagues performed vitrectomy and internal limiting membrane (ILM) peeling in patients with ERM using a vitrectomy surgery robot, under general anesthesia [27]. This was the first remotely controlled robot-assisted retinal surgery to be conducted in humans. No obvious adverse events occurred with either the surgery performed by the vitreous surgeon or that performed by the robot, but it took longer for the robot to reach the retinal surface from the port and to raise the ILM flap than it did for the vitreous surgeon. Thus, the history of robotics has centered on “operation systems,” where robots perform surgery instead of surgeons. However, in recent years, “operation assistance systems,” which act as assistants to support surgery, have also been developed [28]. Furthermore, robotic observation systems have been developed to enhance observation during vitrectomy surgery. In summary, the current robotic systems used in vitreoretinal surgery can be broadly classified as operation systems, operation assistance systems, and observation systems [28] (Table 1).

**Table 1:** Robots for vitreoretinal surgery worldwide

Category	Operation system						Operation assistance system		Observation system
System	PRECEYES surgical system	ORYOM system	Intraocular Robotic Interventional and Surgical System (IRSS)	Ophthorobotics AG	RAM!S	OctoMag	MICRON	iArmS	OQrimo
Country/ University・Company	Netherlands/Preceyes B.V, Zeiss・Eindhoven University of Technology	Israel/Forsight Robotics	United States/University of California, Los Angeles	Switzerland/ Ophthorobotics ETH Zurich	Germany/ Technical University of Munich	Switzerland/ ETH Zurich	United States/Carnegie Mellon University, Johns Hopkins University・Robotics Institute	Japan/Denso, Toho-tech	Japan/ Riverfield・ Kyushu University, Tokyo Institute of Technology, Juntendo University, Yamaguchi University
Current stage	Approved as a medical device in the European Union (2019)	Aiming for approval as a medical device	Not yet ready for clinical trials	Patent obtained in 2023; clinical trials to begin in 2024	Not published	Not published	No longer active shown in home page	Launched by Denso (2015)	Approved as a medical device in Japan (2023)
Functions	ILM peeling, subretinal injections	Mainly automatic cataract surgery	Mainly automatic cataract surgery, retinal vein cannulation	Intravitreal injections	Subretinal injections	Retinal vein cannulation	Tremor suppression	Tremor suppression	Endoscope or light pipe holding

## Vitreoretinal surgery robots (Table 1)

### PRECEYES surgical system (https://www.preceyes.nl)

The Preceyes Surgical System has been developed by Preceyes B.V., which was founded by researchers at the Eindhoven University of Technology in the Netherlands. Currently, Preceyes, which owns this system, is operated by Zeiss. This system is for intraocular surgery, especially vitrectomy surgery, and the surgeon operates the robot arm with a joystick [29, 30]. It has the same concept (leader-follower robot) as the da Vinci Surgical System, but there is no operating cockpit, and the surgeon operates by peering into a microscope as usual. Unlike general surgery, the purpose is not remote operation but high precision, which is extremely high at 10 µm [29]. As described above, this group confirmed its usefulness and safety in patients with ERM and subretinal hemorrhage in 2018 [27]. Furthermore, the Preceyes Surgical System was successfully used in patients under local anesthesia. In recent years, a study was conducted using the Eyesi surgical simulator by various surgeons to compare ILM peeling performed using a robot with ILM peeling performed manually. The results showed that although the operation took longer with the robot, less macular retinal hemorrhage occurred [31]. The Preceyes Surgical System was approved as a medical device in the European Union in 2019. In 2021, a first-in-human study of subretinal drug injection using this robot during vitreoretinal surgery under local anesthesia for macular hemorrhage was reported [32].

### ORYOM system (https://www.forsightrobotics.com)

Forsight Robotics’ ORYOM system (Israel) is a highly accurate, controlled ophthalmic surgical robot. The surgeon wears 3D glasses and performs the surgery remotely. Currently, the company is aiming to apply the system to cataract surgery, but it is also considering adapting it to vitreoretinal surgery in the future [33].

### Intraocular robotic interventional and surgical system (IRISS)

The IRISS is an intraocular surgery robot developed by a group at the University of California, Los Angeles. This robot can perform both vitrectomy surgery and cataract surgery. It has 2 independent arms and can use current surgical instruments. The IRISS was first introduced in 2013, and the surgeon holds a joystick and controls it remotely [34]. An experimental study showed that the accuracy of positioning the tool tip was 205 ± 3 µm [35]. In recent years, an optical coherence tomography (OCT)-integrated robotic system has been developed and tested on ex vivo pig eyes [36]. Currently, semiautomation has been achieved mainly in cataract surgery, but it is expected to be applied to vitreoretinal surgery in the future [37].

### Ophthorobotics AG (https://www.ophthorobotics.com)

Ophthorobotics AG is a venture company founded in 2014 by researchers from the Swiss Federal Institute of Technology in Zurich (ETH Zurich). Currently, anti-VEGF therapy has become the first choice for various vitreoretinal diseases and has achieved good visual improvement [38, 39]. As the number of applicable diseases expands, the burden on medical care due to the increase in the number of patients has become an issue [40]. Ophthorobotics AG has developed a robot that safely performs intravitreal injections. This system is operated by a human operator, who places the robot on the patient’s head and automatically recognizes and tracks the pupil to safely perform intravitreal injections via the pars plana. The robot has been successfully tested using porcine eyes, which is expected to reduce medical staff costs [41].

### RAM!S

The RAM!S system is an ophthalmic surgery robot introduced by researchers at the Technical University of Munich in 2013 [42]. This robotic system is extremely small (94 ± 28 x 33.5 x 18.5 mm, 306 g), approximately the size of an average human hand [19]. Furthermore, the tool-tip positional accuracy is 5 µm, and the group has recently evaluated subretinal injections using ex vivo pig eyes [43].

### Octomag

Octomag is a 5-degree-of-freedom (5-DOF) wireless magnetic control of a fully untethered microrobot, introduced in 2010 [44]. This is a fully wirelessly operated robot designed to perform precise tasks inside the eye. A chick chorioallantoic membrane was also used to perform retinal vein cannulation experiments with this robot [45].

### MICRON

The MICRON was developed in 2010 as a collaboration between the Robotics Institute at Carnegie Mellon University and Johns Hopkins University [46]. This robot is a device held by the surgeon, and it suppresses hand tremors. The position of the tool tip is evaluated by surgeons and nonsurgeons, and the error rate was reduced by 32–52% [47]. This reduction suggests that more precise techniques in vitreoretinal surgery can be achieved. Furthermore, this technology will be particularly useful for surgeons with little experience, potentially making vitreoretinal surgery available at more facilities. However, the project’s homepage now states that it is no longer active (https://www.ri.cmu.edu/project/micron-intelligent-microsurgical-instruments/).

## Assistant robot: stabilizing the surgeon’s arm and quantifying its movement, iArmS (https://www.toho-tec.co.jp/products/medical/iarms/)

Departing from conventional development methods, Enaida and colleagues customized the passive surgical support robot iArmS (Intelligent Arm Support System), a commercially available nonmedical device specifically for ophthalmic surgery to stabilize the surgeon’s arm [48]. Passive robots, lacking motor-driven components, eliminate the possibility of malfunctions and provide passive assistance to the operator’s maneuvers. Surface electromyography measurements were conducted to evaluate fatigue levels during surgical simulations with and without the device to assess its efficacy [49]. Furthermore, its performance was evaluated in addressing challenges such as ILM peeling in vitrectomy. Results suggested the device’s capability to alleviate intraoperative muscle fatigue and reduce hand tremor [48, 49]. Subsequently, actual surgeries were performed using the prototype, and clinical evaluations were undertaken to assess its effectiveness in real-world scenarios.

## Assistant/Observation robot: endoscope-holding robot, OQrimo (https://riverfieldinc.com/en/)

### Endoscopic vitrectomy

Intraocular observation is one of the important elements in vitrectomy surgery. Unlike the currently mainstream wide-viewing systems (top-down/bird’s eye), intraocular endoscopes are used for side-on observation and are advantageous for peripheral observation because they are inserted through a trocar port [50, 51]. The first intraocular endoscope was developed in 1934, before the advent of vitrectomy surgery [52]. The first intraocular endoscope was large, with a diameter of 6.5 mm, and was used for the purpose of removing intraocular foreign bodies [53]. Later, in 1981, Norris and colleagues developed a 1.7 mm-diameter intraocular endoscope for the purpose of removing intraocular foreign bodies [54]. With the development and advancement of vitrectomy, intraocular endoscopes have evolved for peripheral observation, and their usefulness in cases of corneal or lens opacity has been reported [50, 55]. Eguchi and colleagues developed a 20-gauge (20G) electronic video endoscope system for intraocular surgery [56]. Currently, intraocular endoscopes with high resolutions of 23G, 25G, and even 27G are available on the market. However, their use in vitrectomy is limited because they require the surgeon to hold the endoscope with 1 hand and it is difficult to recognize its location inside the eye, so there is a learning curve to master its use for vitrectomy surgeons.

### Development process of OQrimo

**Fig. 2:**
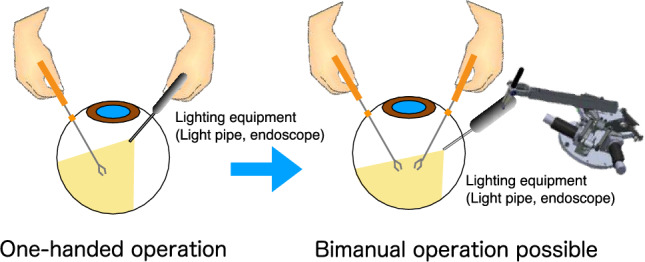
Intraocular endoscope and intraocular endoscope-holding robot in vitrectomy. In vitrectomy, the use of an intraocular endoscope requires the surgeon to perform 1-handed operation. However, the development of the intraocular endoscope-holding robot allows the surgeon to perform bimanual operation

From the beginning, our team thought of making an “operation assistant robot” or “observation robot” rather than an “operation robot” (Table 1). Apart from the importance of an “operation robot,” as mentioned above, to establish technology to safely operate intraocular tools, it is more realistic to develop an “operation assistant robot” as the first development task. In surgeries in other areas, multiple assistants, such as scopists with intraocular endoscopes, support the surgeon. However, in vitreoretinal surgery, especially during the intraocular operation, assistants have almost no role owing to the limitations of the surgical field. In most cases, vitreoretinal surgeons hold lighting equipment (e.g., light pipes and endoscopes) in 1 hand (Fig. 2). Therefore, the essential surgical procedures are almost always performed with the other hand but not with both hands. We have become accustomed to procedures performed with 1 hand, but if both hands can be used in vitreoretinal surgery, there would surely be benefits and it may be possible to develop better surgical procedures (Fig. 2).

From this perspective, we aimed to create a robot that can safely hold an intraocular lighting device and illuminate the area the surgeon wants to see. We targeted light pipes and intraocular endoscopes as lighting devices. The intraocular endoscope is a lighting device, and at the same time, as its resolution improves in the future, it has the potential to become a useful tool when used in conjunction with a noncontact wide-viewing system. Because no other ophthalmic surgery robot based on this idea has been developed, a robot based on this concept is unique and highly original.

The development of an intraocular endoscope-holding robot began in 2012 with the collaboration of Kyushu University, Tokyo Institute of Technology, Juntendo University, Yamaguchi University, and Riverfield Co., Ltd. The research and development of this product accelerated with subsidies from the Japan Agency for Medical Research and Development (AMED) in 2015 and 2017. Figure 1 shows the progress of its development since 2017. With 6 basic and related patents, we consulted with the Pharmaceuticals and Medical Devices Agency (PMDA) before commercializing the product. On April 13, 2023, the product was approved for sale as a general medical device, and a press release was issued by all the collaborating institutions involved in its development.

**Fig. 3:**
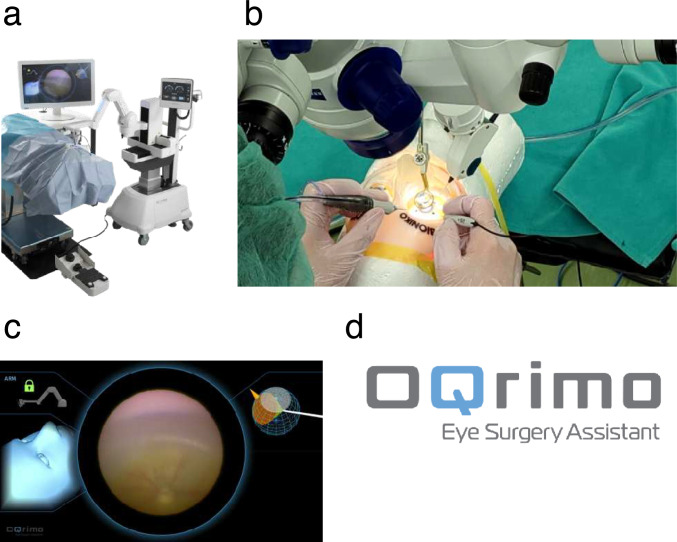
Intraocular endoscope-holding robot OQrimo. The surgeon can operate OQrimo using a foot switch while viewing the endoscope screen for the bimanual manipulation. **a** Overview, **b** Surgical field (using OQrimo with a wide-viewing system), **c** Navigation screen including endoscopic images, **d** Logo mark

The product name is OQrimo (Fig. 3) [28]. The name is derived from the Latin words oculus, meaning eye, and rimor, meaning explorer, because of its explorer-like feature of illuminating the entire inside of the eye. The arm has a gimbal structure, and while the intraocular lighting device is held, it can be freely guided to the intended position by use of the foot controller (Fig. 3a) [57]. The OQrimo holds the endoscope, allowing the surgeon to operate with both hands (Fig. 3b). It also has an intraocular mapping screen, allowing intuitive operation (Fig. 3c). Figure 3d shows the logo, and OQ represents both eyes, and the Q represents the lighting device being inserted into the eyes.

The OQrimo has overcome the first hurdle of commercialization, but rather than immediately releasing it to the market, we plan to introduce it gradually while establishing a safety system. Its initial use will be carried out mainly by ophthalmologists who have been involved in its development, and a “Proper Use Manual” will be created that describes the indications and optimal procedures. After that, ophthalmologists will undergo hands-on training and a license will be issued. In addition, a “physician-led research group” will be set up without the involvement of companies to monitor safety. It will be a while before the product becomes available to the public, but we would like to disclose the information thoroughly and move forward while receiving various opinions.

### The future outlook of OQrimo

The use of OQrimo may change the future of vitreoretinal surgery. By incorporating OQrimo into surgery, surgeons will be able to operate with both hands, which will enable more precise surgery in the treatment of the fibrous membrane in proliferative retinopathies such as PVR and PDR. Furthermore, the assistant robot may be particularly useful in surgery for anterior PVR. It may also enable surgery on the ciliary body, which cannot be observed with the currently available wide-viewing systems. In highly myopic eyes, precise macular manipulation is important for foveoschisis and macular hole. Therefore, placing the trocar further away from the limbus shortens the distance from the macula. Recent reports have shown a method for determining the trocar position using anterior segment OCT before surgery. However, there are facilities that do not have anterior segment OCT, and it cannot be denied that the distance may change after cataract surgery. We have recently reported a method for determining the trocar position using an intraocular endoscope during surgery. Furthermore, this method may support more accurate surgery by allowing direct viewing of the ciliary body, such as intraocular lens fixation in the sclera.

With the information from the intraocular endoscope held by OQrimo, it is possible to constantly monitor the inside of the eye during surgery such as for measurement of the position of the instruments and the distance from the retina [58]. It may be possible to quickly detect the formation of new retinal tears, retinal bleeding, and signs of low intraocular pressure during surgery. Furthermore, since the intraocular endoscope not only has a light source but also observation capabilities, concepts such as automatic tracking of the vitreous cutter are also proposed.

## Prospects

### Artificial intelligence (AI) for robotic vitreoretinal surgery

**Fig. 4:**
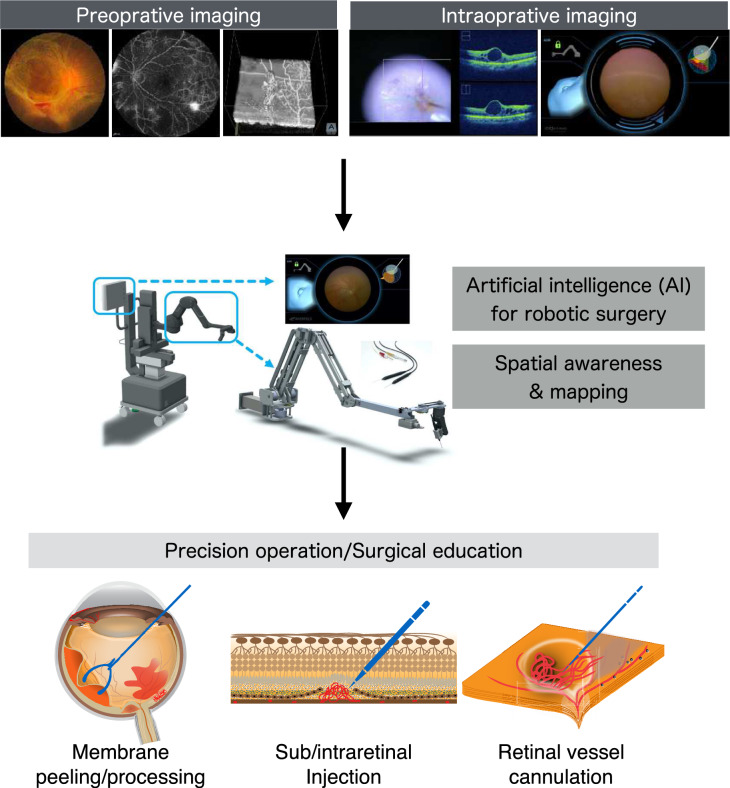
The future of vitreoretinal surgery combined with robotics. Preoperative and intraoperative imaging, such as fundus photography and optical coherence tomography (OCT), will enable precise operation and surgical education for robots using artificial intelligence (AI). Furthermore, spatial awareness and mapping will help improve intraoperative safety. This will allow precise removal and processing of the internal limiting membrane (ILM) and fibrovascular proliferation membranes (in a preoperatively determined shape and direction), precise subretinal and intraretinal injections for gene and cell therapy, and even retinal vessel cannulation for the anticipated custom-made (tailor-made) surgery

The use of AI in the medical field is currently spreading rapidly. In the field of ophthalmology, AI-based image diagnosis using fundus photography and other imaging devices is already being used as a medical device [59]. In recent years, the use of AI in surgery has been expected. Examples of the use of AI in surgery include preoperative and intraoperative diagnosis, intraoperative decision support, surgical education, and postoperative management [60]. The use of AI in robotic vitreoretinal surgery is expected to have the following impacts both preoperatively and intraoperatively. (1) By using preoperative images such as fundus photography and OCT images, it may be possible to optimize the surgical plan for robotics (Fig. 4). In other words, because robotic surgery is highly accurate, it may be possible to program the control of the robot by using AI before surgery. For example, in intravascular treatment (retinal vessel cannulation) of cases of retinal artery occlusion, the site of occlusion can be identified by using preoperative OCT angiography or fluorescein angiography, and the robot will inject drugs into the identified blood vessel and at the appropriate depth (Fig. 4). (2) Since intraoperative images are always recorded, they may be used to make intraoperative decisions such as the need for laser photocoagulation. For example, in the surgical robot processing of fibrovascular membranes in PDR (Fig. 4), it may be possible to make intraoperative judgments such as to minimize intraoperative bleeding. (3) By using AI to teach the robot the precise operating skill of an experienced surgeon, it may be possible to use it for surgical education (Fig. 4).

### Spatial awareness and mapping

An endoscope-holding robot (OQrimo) allows the surgeon to perform vitreoretinal surgery with both hands. However, the vitreous, where surgical operations are performed, is a limited space, and the inner surface is surrounded by the retina, which is a fragile neuronal thin tissue (thickness, 100–300 µm), so understanding the distance between the endoscope held by the robot and the retina is important for safety. We have developed a real-time fundus reconstruction method [58]. This method uses deep learning to measure the distance between the retina and the tip of the endoscope. By using the information on the distance to the retina in conjunction with the robot’s movements, it is possible to constantly recognize the position of the endoscope within the vitreous cavity. The error in distance was approximately 0.5 mm, and the error in eyeball size was approximately 1 mm. In this way, it is predicted that the combination of AI and robots will enable safer surgery (Fig. 4).

### Utilization of robotics in surgical education

Surgical education is crucial for training the next generation of surgeons. However, it is difficult to quantitatively convey the techniques of experienced surgeons. Robots and virtual reality have been reported to be useful for surgical education [61]. In addition, in depopulated areas with few doctors, providing frequent surgical education is challenging; therefore, remote surgical education will be valuable. Furthermore, it might be imaginable to simulate appropriate procedures in advance with the support of robots. The practice of surgical techniques in ophthalmology requires the quantification of procedures. To this end, the Bionic Eye Surgical Evaluator (Bionic-EyE)—a model specifically designed for ophthalmic surgical training—is being actively researched and developed [62]. The Bionic-EyE integrates a meticulously replicated eyeball module with mechanical and biologic characteristics resembling human eyes, complemented by internal sensors for evaluating trainees’ surgical skills. Using the Bionic-EyE, trainees can repeatedly practice forceps manipulation and promptly receive quantitative feedback on their abilities based on sensor data.

### Clinical applications in the future

Some ophthalmologists may believe that the current vitreoretinal surgical methods have reached a state of perfection. Wide-angle observation systems, trocar closure valve systems, MIVS, and other technologies have made it conceivable to perform vitreoretinal surgery more safely and with less invasiveness than previously. However, some cases, such as PDR and PVR, require surgical time and are difficult to gain visual function even with the current systems (Fig. 1). Currently, no cure is available for retinal degenerative diseases such as retinitis pigmentosa, and gene therapy and regenerative medicine are currently being developed (Fig. 1). These treatments require precise and reliable delivery of gene vectors in gene therapy for retinal diseases. In regenerative medicine, if cells cannot be transplanted reliably, there could be a risk of PVR and other diseases. Robotic surgery may be able to contribute to these unmet needs with its accuracy (Fig. 4). Furthermore, the currently available surgeries lack quantitativeness. For example, even in the ILM peeling technique performed by most vitreoretinal surgeons, there is no quantitativeness in the area or position of the peel. Vitreoretinal surgery robots may enable quantitative surgery at the micro level. Further technological advances are expected to equip surgical robots with force sensors and intraocular imaging, and technological advances may also enable even smaller instruments for vitreoretinal diseases [63, 64]. Furthermore, the simultaneous use of multiple surgical robots, such as the PRECEYES Surgical System and OQrimo, may offer synergistic safety and patient benefits. This could lead to customized treatment for each patient, disease, and pathologic type in the future.

Is it necessary for robots to perform procedures that most vitreoretinal surgeons can complete? In cataract surgery, robots using femtosecond lasers have been developed, making safer and more reliable surgery possible. However, at present the use of robots is limited. This may be because the level of precision achievable only with robotic assistance (for example, CCC of a perfect circle) is not directly linked to the outcome of patient satisfaction. Furthermore, in many countries, it is not possible to charge an additional fee for this, and the cost issue is also a big problem for the medical side. However, a time will undoubtedly come when future technological advances may ultimately establish a favorable benefit-risk ratio for robotic assistance in vitreoretinal surgery. From this perspective, it is essential in the future to quantitatively evaluate the superiority and safety of robots and to demonstrate them with evidence.
